# Transcriptomic Characterization Reveals Attributes of High Influenza Virus Productivity in MDCK Cells

**DOI:** 10.3390/v13112200

**Published:** 2021-11-01

**Authors:** Qian Ye, Thu Phan, Wei-Shou Hu, Xuping Liu, Li Fan, Wen-Song Tan, Liang Zhao

**Affiliations:** 1 State Key Laboratory of Bioreactor Engineering, East China University of Science and Technology, Shanghai 200237, China; qy@mail.ecust.edu.cn (Q.Y.); wstan@ecust.edu.cn (W.-S.T.); 2 Department of Chemical Engineering and Materials Science, University of Minnesota, Minneapolis, MN 55455, USA; phanx247@umn.edu; 3 Research and Development Center, Shanghai BioEngine Sci-Tech Co., Ltd., Shanghai 201203, China; liuxp@bio-engine.com.cn (X.L.); fanli@bio-engine.com.cn (L.F.)

**Keywords:** influenza virus, MDCK cells, transcriptome, host shutoff, anti-viral responses

## Abstract

The Madin–Darby Canine Kidney (MDCK) cell line is among the most commonly used cell lines for the production of influenza virus vaccines. As cell culture-based manufacturing is poised to replace egg-based processes, increasing virus production is of paramount importance. To shed light on factors affecting virus productivity, we isolated a subline, H1, which had twice the influenza virus A (IAV) productivity of the parent (P) through cell cloning, and characterized H1 and P in detail on both physical and molecular levels. Transcriptome analysis revealed that within a few hours after IAV infection, viral mRNAs constituted over one fifth of total mRNA, with several viral genes more highly expressed in H1 than P. Functional analysis of the transcriptome dynamics showed that H1 and P responded similarly to IAV infection, and were both subjected to host shutoff and inflammatory responses. Importantly, H1 was more active in translation and RNA processing intrinsically and after infection. Furthermore, H1 had more subdued inflammatory and antiviral responses. Taken together, we postulate that the high productivity of IAV hinges on the balance between suppression of host functions to divert cellular resources and the sustaining of sufficient activities for virus replication. Mechanistic insights into virus productivity can facilitate the process optimization and cell line engineering for advancing influenza vaccine manufacturing.

## 1. Introduction

Human influenza virus inflicts a tremendous societal cost in terms of mobility and mortality [1]. Its counterpart for poultry, avian influenza virus, also causes huge economic costs in agriculture, and sporadically infects humans [2]. Vaccination remains the most effective protection against influenza virus infection. The vast majority of influenza virus vaccines are produced using pathogen-free embryonated chicken eggs, for which the manufacturing requires a well-managed supply chain of embryonated eggs and lacks surge production capacity. Cell culture-based processes are amenable to scale-up and rapid expansion of production capacity in a pandemic [3]. During past decades, the cell culture process has been increasingly used to meet global demand for the seasonal influenza virus vaccine. There has also been a strong interest in enhancing the productivity of the influenza virus in cell culture processes. Efforts include the adaptation of producing cell lines in suspensions instead of adhering them to microcarriers or flask surfaces [4,5], the employment of means to increase cell concentrations [5,6,7,8], adoption of real time monitoring of virus infection dynamics [9], and development of multiscale models for process intensification [10].

The cell line used in the production of the influenza virus hugely influences productivity [11], which hinges on the interplay of several factors including the match of viral surface antigen cell surface receptors, the speed and extent at which the virus can divert cellular functions of the infected cell towards virus replication, the capability of the producing cell to mount an effective anti-viral response, and the propensity of the virus to escape the antiviral response. With the complexity of factors affecting virus productivity, it is not surprising that only a small number of cell lines such as Madin–Darby Canine Kidney (MDCK), Vero, and HEK293 cells are commonly used in influenza A virus (IAV) production. Despite their importance in IAV production and the implications on our capability for rapid responses to pandemics, there has been few studies on the dynamics of cellular behavior in these production cell lines after IAV infection. Host response to virus infection has been a very active area of research due to its interest to public health. Transcriptomic responses to the infection process in host organisms [12], target tissue cells [13,14,15], and in MDCK cell lines [16] have been investigated; yet, their focus has been in the context of infectious disease. There have been few studies on how a production cell line responds to IAV infection, and how the alterations in cell responses affect IAV production.

In this study, we took advantage of the heterogeneity of an MDCK cell line and isolated a cell clone which had enhanced IAV productivity compared to the parental population, and analyzed its transcriptome profile to better understand the attributes which contribute to its superior virus production characteristics. These results paved the way for a better understanding of the cellular mechanisms of higher viral productivity, and provided insight into the process optimization, cell engineering, and construction of superior cell lines for influenza vaccine manufacturing.

## 2. Materials and Methods

### 2.1. Cells, Virus, and Reagents

The MDCK cells (ATCC, CCL-34) were cultivated in a T-flask in Dulbecco’s Modified Eagle Medium (DMEM) (BioEngine Sci-Tech, Shanghai, China) supplemented with 10% (*v*/*v*) fetal bovine serum (FBS) (Biosun, Shanghai, China) at 37 °C in a humidified incubator with 5% CO_2_. Influenza A virus (A/chicken/Guangxi/SIC6/2013(H9N2)) was generously provided by Guangdong Wens Dahuanong Biotechnology Co., LTD (Yunfu, China). The ammonium pyrrolidinedithiocarbamate (PDTC) was from Beyotime Biotechnology (Shanghai, China).

### 2.2. Single Cell Cloning

The cells were seeded in 96-well plates at 0.5 cells per well. Surviving clonal cells in individual wells were picked and expanded. Triplicated cultures of each clone were used for cell counting, virus infection, and maintenance.

### 2.3. Virus Infection

The titer of the seed virus utilized was determined by the 50% tissue-culture-infective dose (TCID_50_) assay on MDCK cells. Multiplicity of infection (MOI) was calculated based on the TCID_50_ titer. Infection was carried out in DMEM, supplemented with 5 μg/mL (~4 BAEE U/mL) TPCK-trypsin (Sigma-Aldrich, Darmstadt, Germany).

For assessment of virus productivity of clonal cells, confluent cells in 96-well plates or T75 flasks were washed twice with phosphate-buffered saline (PBS) before infection at a MOI of 0.01 at 37 °C in a 5% CO_2_ atmosphere. The supernatant was harvested at 72 hpi for HA assay.

For synchronized single-cycle infection, sub-confluent cells in T75 flasks were washed twice with PBS and infected at a MOI of 10 and rocked every 10 min for 1 h. Thereafter, the supernatant was removed, and cells were washed twice with PBS before fresh DMEM were added to resume incubation. For virus release kinetics, 5 mL of medium were harvested, and 5 mL fresh DMEM added every 4 h. Secondary infections were minimized by eliminating trypsin. Aliquots of supernatants were stored at −80 °C until virus titration. Cells from triplicate runs were harvested for transcriptome analysis, mitochondrial membrane potential and apoptosis detection.

### 2.4. Virus Quantification

The hemagglutination assay was performed as described [17]. Total virus particle concentration was determined by assuming that agglutination occurs up to a dilution in which the amount of virus particles equals the number of erythrocytes [18]. Thus, the calculation was based on HA titer and cell concentration of the erythrocyte suspension (2 × 10^7^ cells/mL). A simplified HA assay was used for clonal cells in 96-well plates with a smaller sampling and erythrocyte quantity of 25 μL and a higher concentration of erythrocyte suspension (5 × 10^7^ cells/mL). The last dilution that showed complete hemagglutination was considered the end point. Titers of the HA assay were expressed as log2 HA units per test volume (log2 HAU/100 or 25 μL).

The TCID_50_ assay was performed as described [19]. To identify influenza virus-positive wells, the hemagglutination assay was performed instead of direct observation of the emergence of the CPE (cytopathic effect). The infectious virions per milliliter were calculated using the Reed–Muench method.

### 2.5. Digoxigenin Sialic Acid (SA) Detection Assay

Digoxigenin sialic acid (SA) is detected according to the manufacturer’s instructions for the DIG glycan differentiation kit (Roche, Mannheim, Germany). Briefly, 1 × 10^6^ cells from the parent, high producer 1/2 (H1/H2), and low producer 1 (L1) were collected and stained with DIG labeled MAA for 1 h and washed twice by PBS supplemented with 5% FBS. Cells were analyzed using flow cytometry after incubation with anti-DIG FITC in 37 °C for 1 h.

### 2.6. Analysis of Apoptosis during IAV Infection

After being washed with cold PBS, MDCK cells were incubated with FITC-annexin V and PI following the manufacturer’s instructions (BD Biosciences, Franklin Lakes, NJ, USA). The percent rate of apoptosis was analyzed using CytoFLEX flow cytometry (Beckman Coulter, Brea, CA, USA).

### 2.7. RNA Sequencing by Illumina HiSeq and Transcriptome Analysis

Total RNA extraction, RNA quantification and quality control, mRNA-based library preparations, and next generation sequencing were conducted by GENEWIZ (Suzhou, China). Sequencing was carried out using a 2 × 150 bp paired end configuration with >5G each sample in total.

Sequence reads were mapped with HISAT2 [20] against the genome assembly from the Canis lupus familiaris (GCA_000002285.2 CanFam3.1) and IAV (A/chicken/Guangxi/SIC6/2013 (H9N2) downloaded from the influenza research database (https://www.fludb.org, accessed on 31 March 2020) [21]). The transcript counts of mapped reads for each sample were quantified using featureCounts [22]. Differential gene expression analysis was performed using DEseq2 [23]. Differentially expressed genes (DEGs) were described as significantly upregulated or downregulated if they showed an absolute fold change (FC) is equal to or greater than 2 (|log 2 − FC| ≥ 1), and adjusted *p* value (*p*_adj_) < 0.05 according to DEseq2. The resulting DEGs were analyzed using gene ontology (GO) enrichment analysis (http://geneontology.org/, accessed on 28 October 2020) for biological process domains. Gene set enrichment analysis (GSEA) [24] was performed on the normalized data to identify significantly enriched gene sets (|normalized enrichment score (NES)| > 1.6, FDR < 0.05). The enriched pathways were further plotted using the ggplot2 package in R [25]. The IRF7 genes were annotated using the most recently updated genome annotation (GCF_014441545.1_ROS_Cfam_1.0).

### 2.8. Mitochondrial Protein Separation, Quantification, and Mitochondrial Membrane Potential (MMP) Assay

Cell samples were collected, and the mitochondrion was isolated using a Cell Mitochondria Isolation Kit (Beyotime Biotechnology, China), according to the instruction manual. Mitochondrial protein and total cellular protein were quantified using a BCA Protein Assay Kit (Beyotime Biotechnology, China). Mitochondrial membrane potential was measured according to the manufacturer’s instructions for the mitochondrial membrane potential assay kit with JC-1 dye (Beyotime Biotechnology, Shanghai, China).

### 2.9. Statistical Analysis

Except for the host transcriptome data analysis, the results are shown as the mean ± standard deviation (SD), with at least three separate experiments for each study. Statistical significance was evaluated using the Student’s *t*-test with one-way ANOVA when data could be assessed as normally distributed. A value of *p* < 0.05 was considered statistically significant.

## 3. Results

### 3.1. Isolation of the High IAV-Producing Clone

**Figure 1:**
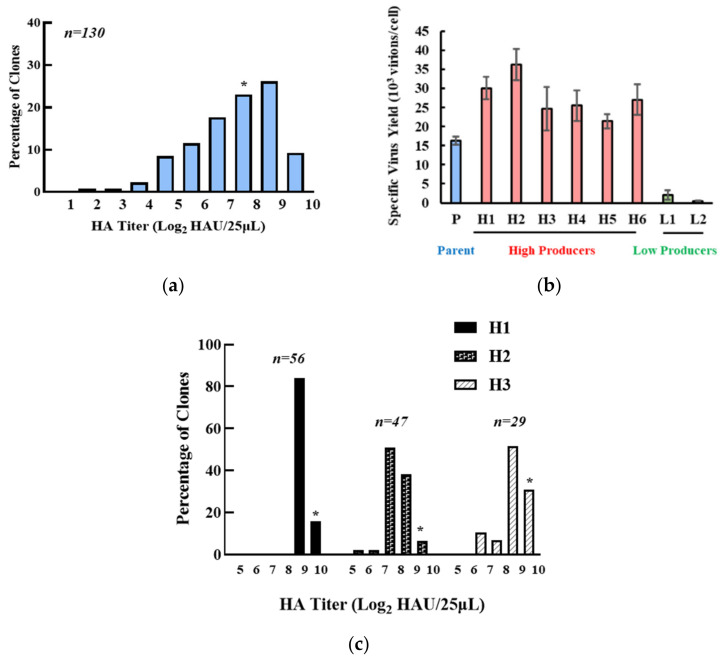
Clonal variation of parental MDCK cells. (**a**) clonal HA titer distribution of parental MDCK cells. (**b**) specific virus yield of selected high- or low-producing clones. Red: High-producing clones, blue: Parent cells, green: Low-producing cells. Experiments were done in triplicated infections. (**c**) HA titer distribution of H1, H2, and H3 subclones after an 80-day passage. * indicates HA titer of the pool.

A total of 130 MDCK cell clones were isolated and assayed for virus productivity, which spread over 200 folds (Figure 1a). The specific virus yields of six high-producing and two low-producing clones as compared to the parent are shown (Figure 1b). Morphologically, the clones and parent were somewhat different (Figure S1a), as reported previously for MDCK cells [26]. However, we did not see any relationship between viral productivity and cell morphology. Avian Influenza viruses preferentially recognize cell receptors containing terminal sialic acid with an α-2,3 (SAα2,3) linkage [27,28]. A lectin staining assay using flowcytometry revealed that the high producers H1 and H2 had comparable levels of SAα2,3 as the parent cells, while the low producer L1 had a lower median than the parent cells (Figure S1b). This data suggests that the low productivity of L1 may be attributed to lower SAα2,3 expression and consequential lower virus infection efficiency, but that the higher titer of the high productivity clones may not be directly related to the SAα2,3 level. The high-producing clones H1, H2, and H3 were passaged for 80 days (~120 population doublings) and then subjected to a second round of single-cell cloning. Most subclones of H1 had HA titers around the level of H1, while titers of some subclones of H2 and H3 fell to levels comparable to P (Figure 1c). The higher productivity in H1 as compared to P was seen over a wide range of MOIs (Figure S3). H1 was then chosen for further study because of its superior stability and productivity.

### 3.2. H1 Sustained Virus Production Longer in Synchronized Single-Cycle Infection

**Figure 2:**
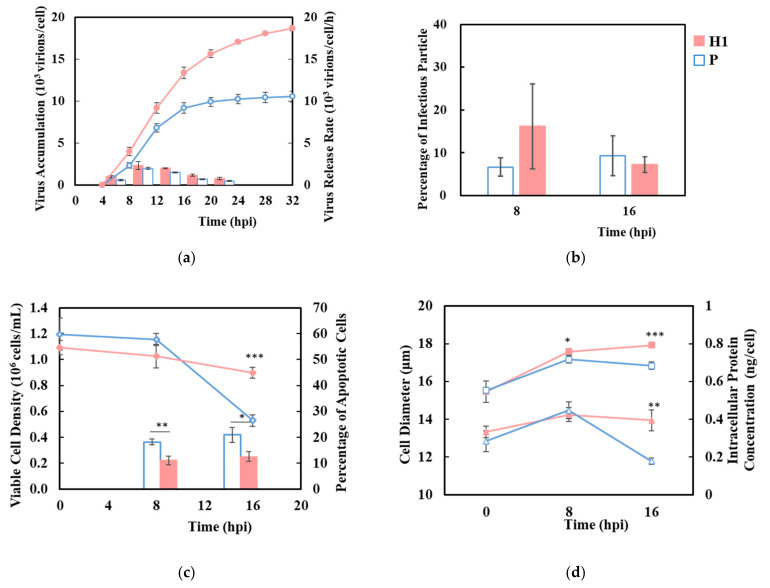
Sustained viability and higher virus production during a synchronized single-cycle infection of influenza A virus in H1 compared to P. (**a**) virus accumulation (circle) and virus release rate (bar), (**b**) percentage of infectious virus to total virions. (**c**) viable cell density (diamond) and percentage of apoptotic cells (bar). (**d**) cell diameter (square) and intracellular protein concentration (triangle). Solid red: H1, hollow blue: P. Experiments were done in triplicated infections. * *p* < 0.05, ** *p* < 0.01, *** *p* < 0.001.

A synchronized single-cycle infection was performed on H1 and P using a high MOI of 10 to ensure that all cells received the virus upon infection [29]. The virus began to emerge in both H1 and P after 4 hpi, reaching a plateau by 16 hpi when the viability became low in P, while continuing at a slower rate until 32 hpi in H1 (Figure 2a,c). Approximately 19,000 virions were released per cell by 32 hpi in H1, which was almost twice that of P. Both H1 and P reached their highest virus release rate between 8–16 hpi (Figure 2a). The proportion of infectious particles in the total virus was ~10%, as assessed by TCID_50_ for both H1 and P (Figure 2b). The increased HA titer in H1 was not at the expense of infectious virion. After virus infection, cell viability decreased, while the percentage of apoptotic cells, cell diameter, and intracellular protein levels increased in both H1 and P. H1 sustained a high viability for longer and maintained a larger cell size, higher intracellular proteins level and a lower extent of apoptosis until 16 hpi (Figure 2c,d).

### 3.3. H1 Had Higher Viral Transcript Levels

**Figure 3:**
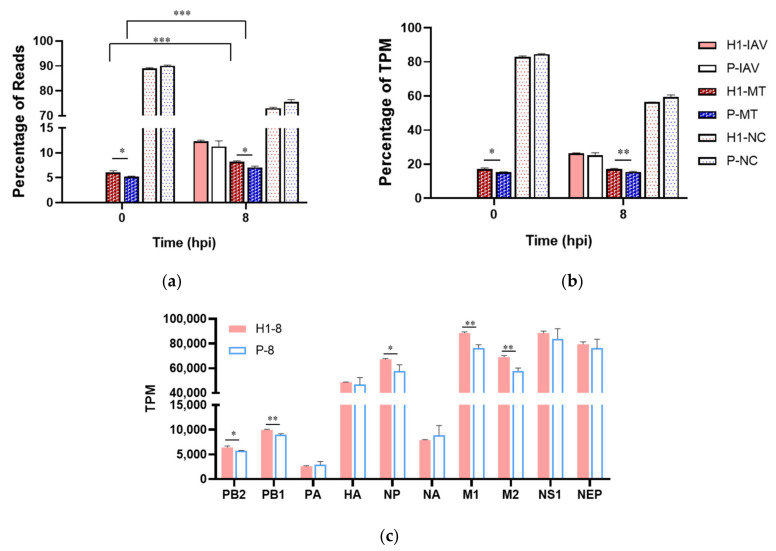
Enhanced expression level of five viral transcripts. (**a**) percentage of reads mapped to viral (IAV), mitochondrial (MT) and nuclear genome (NC). (**b**) percentage of total virus-coded, mitochondrion-coded, and nucleus-coded TPM. (**c**) mRNA level of IAV-coded genes at 8 hpi. Samples from triplicated infections were used for RNAseq data analysis. * *p* < 0.05, ** *p* < 0.01, *** *p* < 0.001.

RNAseq was performed on triplicate cell samples from synchronized single-cycle infections at 0/8/16 hpi for H1 and at 0/8 hpi for P, because of a low viability of P at 16 hpi. The data analysis procedure is described in Figure S3, the raw expression data of all genes in different conditions is listed in Table S6. By 8 hpi, viral mRNAs constituted >10% of all mapped reads (i.e., 10% of total mRNA by mass) or >25% of total mRNA TPM (transcripts per million; i.e., by total transcript molecules) for both H1 and P (Figure 3a,b). Eight out of the 20 top highly expressed transcripts at 8 hpi were viral genes, including all top five, and the rest of the top 20 were from mitochondria genome-coded genes (Table S1). For both H1 and P, the viral mRNAs were more abundant than all the mitochondria-encoded mRNAs combined (Figure 3a,b).

Five out of ten viral transcripts, PB2, PB1, NP, M1, and M2, were expressed at significantly higher levels in H1 than P (Figure 3c). M1 encodes matrix protein, the most abundant among all the structural proteins of IAV, and M2 encodes proton channel protein. NP protein binds to viral genomes and transcripts to form the ribonucleotide protein (RNP) complex and stabilizes the genome. PB1 and PB2, together with PA, form the RNA-dependent RNA polymerase (RdRp). The substantially higher levels of these viral transcripts likely contribute to the higher virus titer.

### 3.4. Parallel Functional Responses of H1 and P to IAV Infection

IAV infection gave rise to large number of differentially expressed genes (DEGs) in both H1 and P (Figure S4a–d). A plot of log2 ratios of normalized counts from DEseq2 of all DEGs between H1–8 and H1–0 against that of P1–8 and P–0 shows that the vast majority of DEGs caused by IAV infection changed in the same direction between H1 and P (Figure S5a). We also compared the transcriptomes of H1–16 hpi and H1–0 and plotted the data against H1–8/H1–0 (Figure S5b); most of the DEGs continued the trend from 8 hpi to 16 hpi.

**Figure 4:**
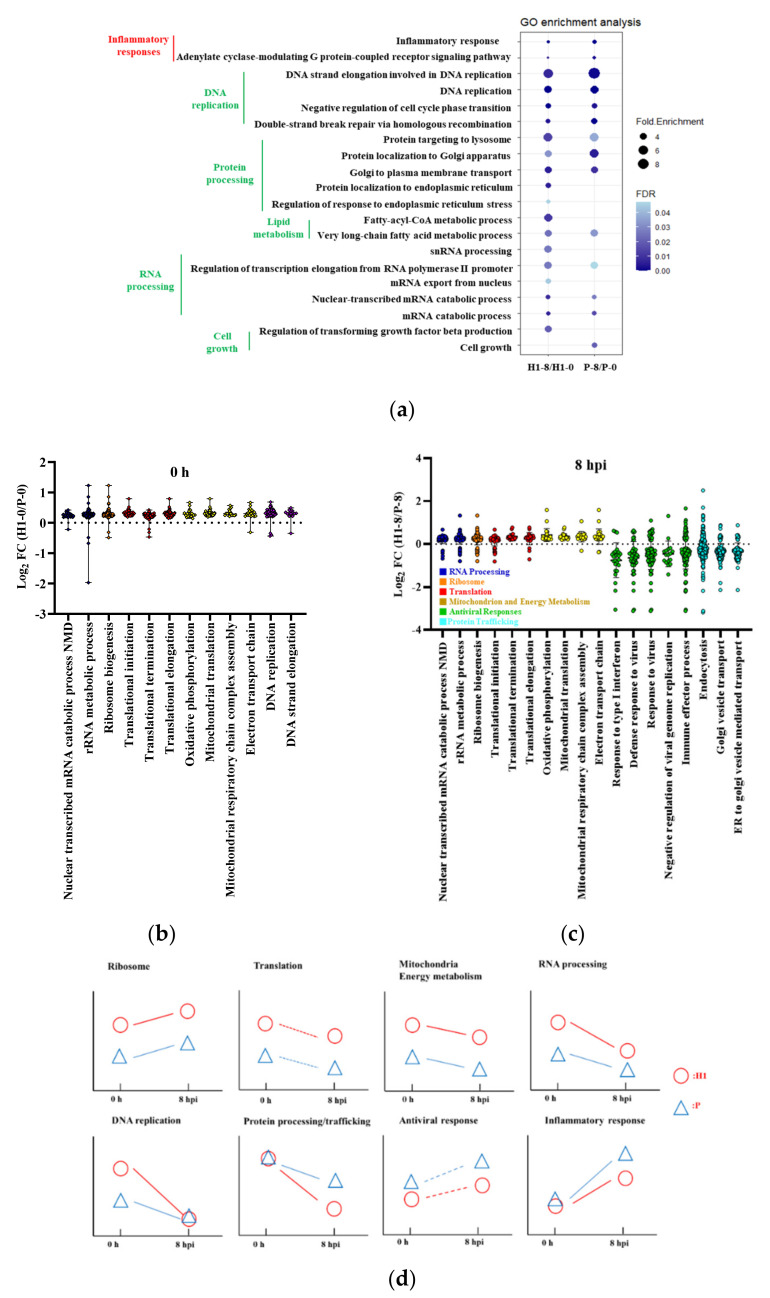
Dynamics of cell response to IAV infection. (**a**) Gene ontology enrichment analysis of host responses of H1 and P upon IAV infection; size represents the fold enrichment, color represents the FDR. Colors of clustered GO terms represents direction of change—red: up−regulated, green: down−regulated. (**b**) transcript ratio of H1 to P in enriched functional classes at 0 h. (**c**) transcript ratio of H1 to P in enriched functional classes at 8 hpi. (**d**) dynamics of enriched functional classes in IAV infection. The relative positions of H1 (red circle) and P (blue triangle) at 0 h and 8 hpi were located according to the GESA results in Figure 4(**b**,**c**) and Table S4. The dynamic changes from 0 h to 8 hpi were inferred either from the transcriptome analysis results (solid line) from GO and GSEA enrichment in Figure 4**a** and Tables S2 and S3, or by inference (dashed line). Samples from triplicated infections were used for RNAseq data analysis.

To identify gene sets enriched upon IAV infection, we conducted pathway analysis using gene ontology (GO) enrichment analysis with DEGs as the input genes and gene set enrichment analysis (GSEA) with the entire transcriptome as input genes. The genes constituting a functional class or gene set used in the two methods were not identical but bore very high degree of similarity. Thus, the enriched functional classes identified by the two methods were often similar, but not identical. The similarity of the transcriptomic responses of H1 and P to IAV infection was reflected by the similar enriched functional classes identified by GO analysis (Figure 4a, Table S2) and GSEA (Figure S5c, Table S3). The enriched functional classes commonly identified by both GO analysis and GSEA fell into a few functional class clusters: DNA replication, protein processing and trafficking, lipid metabolism, and RNA processing, which were downregulated. Additionally, GO analysis identified inflammatory response and G-protein signaling as upregulated and cell growth and lipid metabolism as downregulated upon infection, while GSEA identified ribosomes as upregulated.

The results show that H1 and P responded to IAV infection in a very similar fashion. Hence, the genetic attributes for the differential virus productivity between H1 and P are likely to be in their different degrees of expression changes in response to infection. That the degree of expression change is somewhat different between H1 and P, can be seen in these genes’ positioning at some distance from the x = y line (dashed line) in Figure S5a.

### 3.5. Identifying Differentially Expressed Functional Classes between H1 and P

To further explore possible gene expression changes that may confer a high IAV yield on H1, we compared functional class enrichment between H1 and P before and after infection. The extent of differential gene expression between H1 and P was small, both before and after infection (Figure S4e–g). Gene ontology did not identify any relevant pathways in H1/P comparisons, while several were identified by GSEA (Table S4). The expression fold-changes of genes with *p*_adj_ < 0.05 in the enriched gene sets were gathered in Figure 4b,c. Interestingly, several of the enriched functional groups (RNA processing, DNA replication and protein trafficking) overlapped with those enriched in post- and pre-infection comparison. Additionally, the mitochondria group (Table S4) overlapped with the energy metabolism group detected from the pre- and post-infection comparison (Table S3). The majority of DEGs in these functional classes were expressed moderately higher in H1 than in P. The gene set group of DNA replication was enriched only in 0 h (Table S4). An additional group of genes related to the host cell antiviral response became enriched at 8 hpi. Functional enrichment analysis thus indicated that the genes whose expression differentiated H1 from P were also those which responded to IAV infection.

### 3.6. Differential Functional Enrichment in H1 Versus P Comparisons

Since the enriched functional clusters across both time (0 h vs. 8 hpi) and cell line (H1 vs. P) were highly similar, we plotted the dynamics of their relative levels in Figure 4d. Four functional clusters, including ribosome, translation, mitochondrial-related functions, and RNA processing had intrinsically higher activity in H1, and remained higher after infection. DNA replication was more elevated in H1 initially, but the difference with P diminished at 8 hpi. In terms of time dynamics, RNA processing, DNA replication, translation, and protein processing/trafficking, all were downregulated after infection as part of host shutoff. In contrast, the inflammatory response and antiviral response were both upregulated upon infection. These two functional groups also differed from most other groups in that the elevation of their activity was more vigorous in P than in H1.

#### 3.6.1. Elevated Mitochondrial Activities in H1

**Figure 5:**
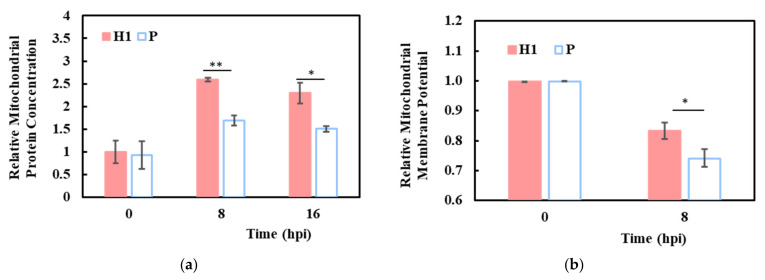
Elevated mitochondrial activities in H1 compared to P. (**a**) relative mitochondrial protein concentrations in H1 and P. (**b**) relative mitochondrial membrane potential in H1 and P. Experiments were done in triplicated infections. * *p* < 0.05, ** *p* < 0.01.

The higher mitochondria-related functions at 0 h and 8 hpi were corroborated by increased mitochondrion-encoded transcripts in H1 than in P, both before and after infection (Figure 3a,b). This was further supported by a higher total mitochondrial protein content (Figure 5a) and mitochondrial membrane potential (MMP; Figure 5b) in H1 at 8 hpi. Despite the general downward trend of mitochondria/energy and lipid metabolism-related functional groups (Figure 4d, Table S3), H1 maintained its higher activities—possibly contributing to its higher virus productivity.

#### 3.6.2. Subdued Inflammatory and Antiviral Response in H1

**Figure 6:**
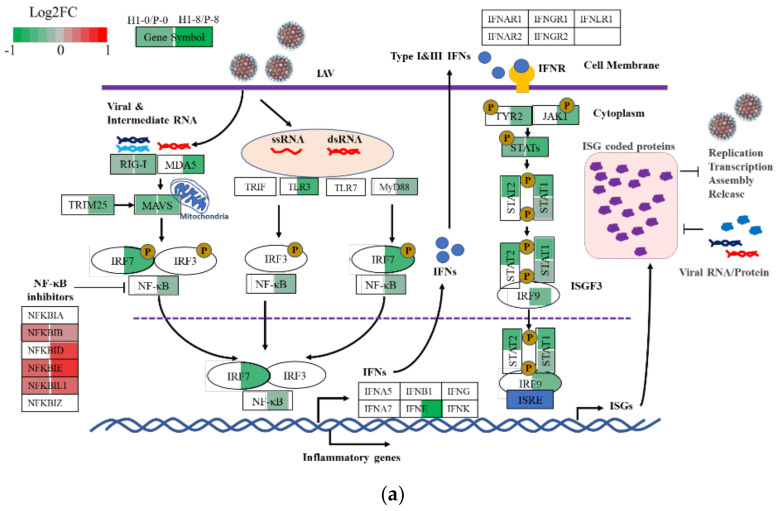

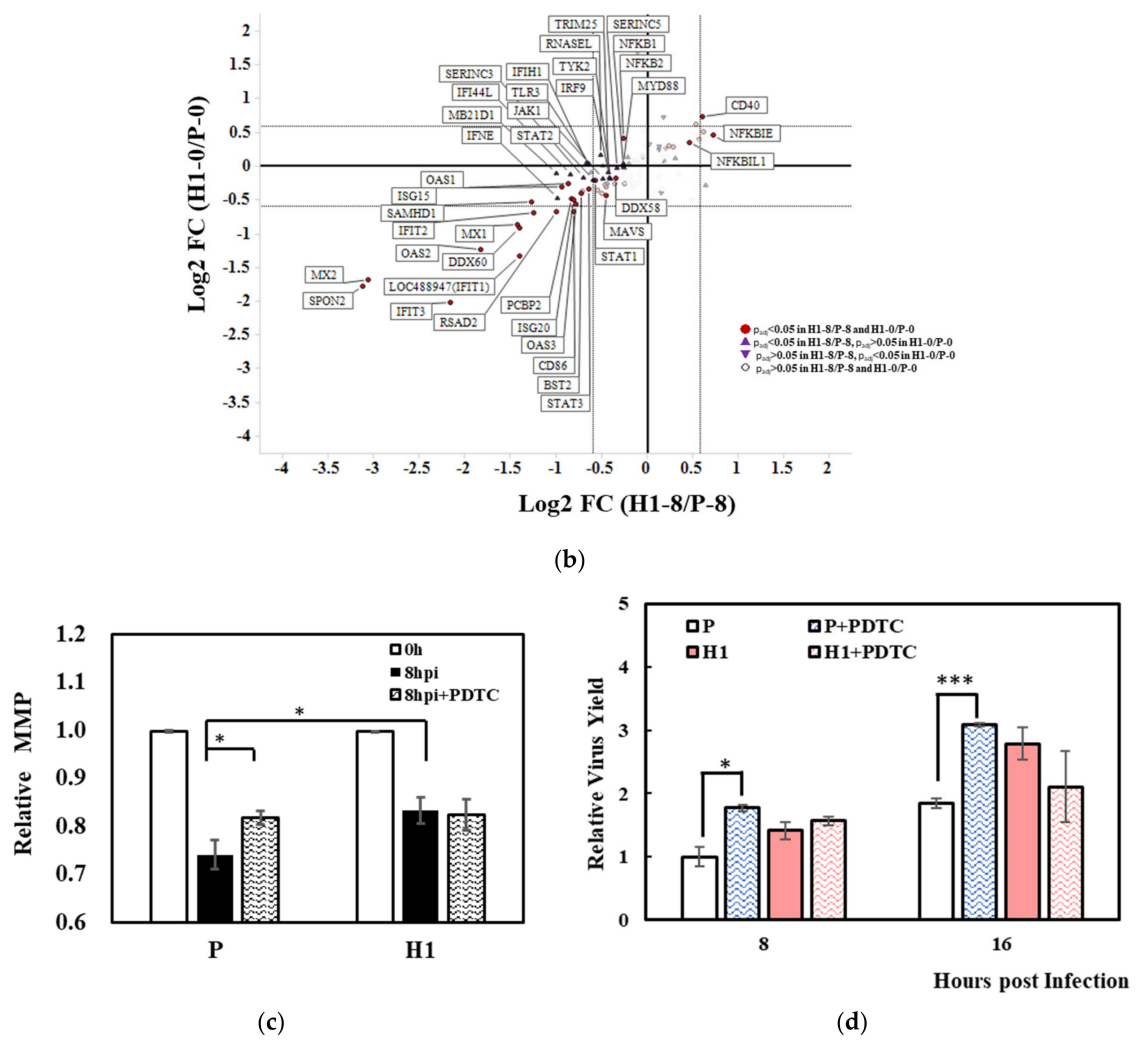
Reduced defense response to virus in H1 compared to P. (**a**) comparison of gene expression level of H1 and P in innate immune response against IAV infection at 0 h and 8 hpi. Each box represents a gene, different colors represent the Log2FC of H1/P (red: upregulated with *p*_adj_ < 0.05, green: downregulated with *p*_adj_ < 0.05, white: *p*_adj_ > 0.05 or average TPM < 1), colors shown in the left of the box represent the comparison at 0 h; in the right they right represent the comparison at 8 hpi. Diagram adapted from Chen, et al. [30] (**b**) comparison of expression levels of anti−viral genes or ISGs in H1 and P before and after virus infection (purple triangle represents genes with *p*_adj_ < 0.05 in comparison H1–0/P–0, inverted triangle represents genes with *p*_adj_ < 0.05 in comparison H1–8/P–8, red circle represents genes with *p*_adj_ < 0.05 in both comparisons). (**c**) effect of PDTC addition in H1 and P on mitochondrial membrane potential. (**d**) effect of PDTC addition in H1 and P on specific virus yield. Experiments were done in triplicated infections. * *p* < 0.05, *** *p* < 0.001.

IAV infection elicits inflammatory and antiviral responses in the host cell; the responses were more vigorous in P than in H1. Antiviral activity is part of the cell’s innate immune response. Upon virus infection, the non-self RNAs are detected by RIG-I or TLRs, leading to the activation of downstream transcription factors IRFs and NFκBs and their translocation to the nucleus, where they trigger the production of interferons and inflammatory cytokines (Figure 6a). The secreted interferon binds to cell surface interferon receptors (IFNRs) and activates the JAK-STAT signaling pathway that leads to the synthesis of ISGs (interferon stimulated genes) [30]. These ISGs have important antiviral activities which inhibit various steps of influenza virus propagation [30,31,32]. The IRF7 (TPM = 63.21 ± 4.78) and the upper stream genes were mostly downregulated in H1 compared to P at 8 hpi. Many genes in the NFκB network and components of NFκB1 and NFκB2 were expressed at lower transcript levels (green, q < 0.05) in H1 than P, whereas most of the NFκB inhibitor genes were at higher levels in H1 (red; Figure 6a and Table S5).

The transcript changes in interferon genes and ISGs downstream of NFκB signaling are shown in Figure 6b. Most ISGs and other viral suppressive genes were expressed at lower levels in H1 than P after infection (Figure 6b, TPM listed in Table S5). Among the few which were expressed at higher levels in H1 were NFκB inhibitors. It is highly probable that the less vigorous inflammatory response and antiviral response in H1 contributed to its high virus productivity.

With our hypothesis that a subdued inflammatory response contributed to the higher IAV productivity in H1, we tested the effect of a moderate suppression of NFκB signaling on IAV production by adding a low level (10 µM) of NFκB inhibitor PDTC (ammonium pyrrolidinedithiocarbamate) during infection. Interestingly, the virus productivity and mitochondrial membrane potential both increased in P but not in H1 (Figure 6c,d). The results corroborated the notion that a subdued NFκB-induced inflammatory and antiviral response favors more efficient virus production.

## 4. Discussion

### 4.1. H1 and P Respond to Infection Similarly but Differ in Their Magnitude

In this work, we exploited the population heterogeneity of MDCK cells to derive high IAV-producing cell clones whose superior virus productivity is stable over long-term culture. In synchronized single cycle infection cultures, the high-producing H1 clone produced twice as many viruses as P, and this higher virus productivity was accompanied by sustaining a high viability for longer. Being mindful that a complex trait like high virus productivity is not likely affected by a single master regulator, but more probably influenced by a plethora of genes, we employed functional enrichment analysis on the RNAseq data to better understand the functional changes in H1 that contribute to its high productivity.

We performed GO analysis and GSEA across two dimensions, comparing before (0 h) and after (8 hpi) virus infection for both H1 and P, as well as comparing H1 to P for both 0 h and 8 hpi. The comparison shows that H1 and P are very similar, and they respond to IAV infection very similarly. The gene sets enriched in the comparison in both dimensions, between cell lines and between infection times, largely fell into the same functional groups (Figure 4), suggesting that genes which differentiate H1 and P largely overlap with those that alter their expression upon IAV infection. Several functional groups that we identified (cell cycle, antiviral defense, respiratory chain/ribosomal protein and mRNA processing) overlap with those described in an IAV infection study on A549 cells using RNAseq and ribosome profiling [33].

Intrinsically, even before infection, H1 had higher ribosome, translation, mitochondria/energy metabolism, RNA processing, and DNA replication activities (Figure 4d). Except for DNA replication, all the other functional groups remained more active in H1 at 8 hpi. All of these groups may contribute to higher robust growth, prolonged viability, and increased productivity. The enrichment of mitochondria-related gene sets in H1 was accompanied by elevated levels of mitochondrial genome-coded transcripts (Figure 3a,b), and higher protein content and membrane potential (Figure 5a,b). After infection, DNA replication activity decreased in both H1 and P (Figure 4d). Virus infection often causes inhibition of host cell replication, which suppresses the reactions of host macromolecule synthesis. Several IAV proteins interact with host cell proteins to trigger cell cycle arrest; for example, M1 protein interacts with host DNA replication protein complex GINS, which is a crucial component in the initiation of DNA replication, to block the host cell cycle [34]. Suppression of DNA replication after infection, as identified by functional enrichment analysis, was thus expected. Furthermore, it is plausible that the more active DNA replication in H1 before infection helped support the longer viability seen in its production culture and contributed to its high productivity.

### 4.2. H1 and P Both Exhibit Host Shutoff but H1 Retained Higher Activities

IAV has the remarkable capability of diverting cellular resources to its own synthesis. Within 8 h after infection, viral mRNA accumulated to a huge proportion of total mRNA. Similar observations of viral dominance at the transcript and protein levels for different IAV strains in different cells have been reported recently [33,35,36,37]. Three IAV structural protein genes, M1, M2 and NP had significantly higher transcript levels in H1 (Figure 3c). For M1 and M2, the difference in abundance levels between H1 and P amounted to more than 10,000 TPM—a huge difference considering the median of the host cell mRNA was only 10 TPM. In addition to its role as a structural protein, M2 has been shown to anchor to mitochondria, promote mitochondria fusion, and increase mitochondria numbers [38]. This might contribute to the higher mitochondrial protein content and MMP seen in H1 after infection (Figure 5a,b).

Virus infection leads to the suppression of host cell transcription and translation, a process considered to be part of virus takeover of cellular functions to divert cellular materials for virus replication and to suppress the expression of cellular antiviral genes in order to avoid the innate immune response [33,39]. The downregulation of RNA processing, translation, and protein processing and trafficking reflects such host cell shutoff. IAV mRNA synthesis by RdRp requires hijacking of the 5′ N7-methyl guanosine (m7G) cap from nascent host mRNA synthesized by host RNA polymerase II [40]. The resulting 5′-cap-less cellular mRNAs are degraded to provide nucleotides for viral RNA synthesis. Viral transcription thus requires nascent host RNA synthesis to supply 5′ end caps, as well as to host transcription suppression for diversion of resources. The accumulation of viral mRNA to such a high level in both P and H1 at 8 hpi thus demands a delicate balance between active synthesis and shutoff. In H1 we saw the RNA processing being shutoff, and we also saw higher activity than in P. It is probable that H1 struck a superior balance to that of P for IAV replication.

Host cell translation shutoff has been seen in the infection of many viruses. For example, poliovirus infection causes protease cleavage of translation initiation factors [41]. Several studies on IAV have shown that shutoff of translation primarily affects host proteins rather than viral proteins through differential translation initiation [39,42]. However, a ribosome profiling study of a different strain of IAV showed similar ribosome loading on viral or host mRNA [33]. It is important to note that viral and host mRNAs in an infected cell compete for translation machinery for protein synthesis. Just by the sheer hyper-abundance of viral transcripts, viral protein synthesis will overtake host protein synthesis. Even under host shutoff conditions, viral protein synthesis continues to run its course. Viral protein production therefore also hinges on the balance of two facets—a negative force of host translation shutoff and a positive effect of viral mRNA hyper-abundance. That the viral mRNAs are even more abundant in H1 than P is likely to have a positive effect on the virus productivity of H1. The fact that transcription and translation activity remained higher in H1 upon host shutoff likely contributed positively to the higher productivity of H1.

### 4.3. H1 Had Subdued Inflammatory and Antiviral Responses

Both the inflammatory response and antiviral response functional groups were prominently upregulated after infection. Their up-regulation has a suppressive effect on virus production. Among the upregulated genes, interferon and inflammatory cytokines are secreted and diffusible, and capable of triggering antiviral responses in infected and uninfected cells. The efficiency of virus replication is a race between interferon production, induction of antiviral genes and the spread of virus infection. The outcome of the race is dependent on the proportion of cells infected initially (i.e., MOI) and the speed of secondary infection. The differential expression of antiviral genes between H1 and P probably contribute to their different sensitivity to MOI, as seen in Figure S2.

A number of DEGs in these groups have been implicated in virus productivity. For example, BST2, also known as tetherin, retains newly formed enveloped virus particles in the cell to prevent their spread [43]. Knockout of BST2 in MDCK and Vero cell lines increased influenza virus release [44]. However, the activity of BST2 was reported to be countered by the IAV M2 [45]. Hence, the IAV productivity-boosting effect of the lower expression of BST2 in H1 might be countered by its higher M2. The complex viral protein and host cell protein interactions need to be further studied. OAS1/2/3 activates RNase L to degrade host and viral RNA [46]. Knockout of OAS1/2/3 increased influenza virus titer [47]. All of these antiviral DEGs were expressed at lower levels in H1 after being upregulated upon infection. Several ISGs, including IFIT1/2 protein and RSAD2, also followed the same pattern of being expressed at lower levels in H1. RSAD2, also known as viperin, restricts the replication of several viruses, including Zika Virus, tick-borne encephalitis virus and measles virus [48,49]. The interferon regulatory factor 7 (IRF7) is known as the master transcription factor of the type I interferon response in mammalian species. Knockdown of IRF7 in MDCK cells or knockout in chicken embryonic fibroblast (DF-1) cells showed increased viral titers [50,51]. The more subdued inflammatory and antiviral response of H1 certainly favors a vigorous virus production.

The NFκB signaling pathway appears to play roles both in facilitating IAV replication through the binding of M2 protein to P60 of NFkB1 [52] and in promoting the inflammatory reaction and antiviral response. Addition of a low level (10 μM) of NFκB inhibitor (PDTC) increased IAV production and elevated MMP only in P, which had a stronger inflammatory response than H1. This result from adding low levels of PDTC did not align with a few previous studies, in which adding high levels of PDTC (50–5000 μM) negatively affected virus production in various cells [52,53,54,55]. The mechanism of the NFκB signaling pathway being essential for IAV replication, as well as for suppressing IAV propagation, warrants further investigation.

Taken together, the functional enrichment analysis supports the notion that the higher IAV production seen in H1 is a complex trait which is brought about by colossal changes in many cellular functions. Intrinsically, the high-producing H1 cell is more active than P in several cellular components and functions, including in ribosomes and mitochondria, and in DNA and RNA processing. These more robust cellular properties were maintained upon infection. We posit that high IAV productivity requires the balance of suppression of host functions for diversion of cellular resources and sustaining of sufficient activities for virus replication. Furthermore, the more subdued inflammation reactions and lower degree of host antiviral reactions in H1 potentially aided in increasing productivity. Importantly, we also showed that naturally there was sufficient genetic heterogeneity in the host cell population for the isolation of cells with altered responses to virus infection and with high virus productivity. The virus-responsive genes from this report and other similar studies constitute a gene candidate pool for engineering superior cell lines for influenza vaccine manufacturing.

## Data Availability

The raw data presented in this study are available at https://doi.org/10.5281/zenodo.5596506, accessed on 7 September 2021.

## Supplementary Materials

The following are available online at www.mdpi.com/article/10.3390/v13112200/s1, Figure S1. (a) Morphology of parental cells and high producing clones. (b) flowcytometric assay of α-2,3 SA receptor in the H1, H2, L1, and parental cells (P). Figure S2. H1 has higher virus productivity at different MOIs. Figure S3. Transcriptome analysis pipeline. Figure S4. Pair-wise comparison gene expression. Figure S5. Comparison of response upon virus infection in H1 and P. Table S1: The TPM of top 20 most abundant transcripts. Table S2: The enriched functional classes upon IAV infection by GO in H1 and P. Table S3: The enriched functional classes upon IAV infection by GSEA in H1 and P. Table S4: The enriched gene sets by GSEA in H1-0/P-0 and H1-8/P-8 comparison. Table S5: Representative differentially expressed genes in antiviral responses. Table S6: Raw data of all the gene expression (Normalized Counts, Average TPM) and DEseq2 output.
