# Supplementary material for: Transcriptomic Characterization Reveals Attributes of High Influenza Virus Productivity in MDCK Cells
Source: Viruses. 2021 Nov 1;13(11):2200. doi: 10.3390/v13112200 (PMC8620111; doi:10.3390/v13112200)
Supplement: Supplementary file 1 [file viruses-13-02200-s001.zip › supplementary correct/viruses-1391821-supplementary-for authors-QYedited (1).pdf]

viruses

Supplementary Materials

Transcriptomic Characterization Reveals Attributes of High Influenza Virus Productivity in MDCK Cells

Table S1. The TPM of top 20 most abundant transcripts.

Rank	Gene Symbol	Description	Average TPM				
			H1-0	H1-8*	H1-16	P-0	P-8
1	IAV-M1	IAV matrix protein 1	0	88695	65993	0	76368
2	IAV-NS1	IAV nonstructural protein 1	0	88392	60070	0	83935
3	IAV-NEP	IAV nonstructural protein 2	0	79468	57491	0	76250
4	IAV-M2	IAV matrix protein 2	0	69210	54393	0	58047
5	IAV-NP	IAV nucleoprotein	0	67307	29166	0	57682
6	MT-COX1	Mitochondrially Cytochrome c oxidase, subunit 1	30013	57279	101864	27630	49648
7	MT-ATP8	Mitochondrial ATP synthase, Fo subunit 8	36261	50936	98383	32275	43588
8	MT-ATP6	Mitochondrial ATP synthase, Fo subunit 6	36721	50135	84189	30996	40968
9	IAV-HA	IAV haemagglutinin	0	48553	97918	0	46906
10	MT-COX3	Mitochondrially Cytochrome c oxidase, subunit 3	29659	42137	71613	23968	35282
11	MT-COX2	Mitochondrially Cytochrome c oxidase, subunit 2	18863	32035	47988	15984	26053
12	MT-ND1	Mitochondrial NADH dehydrogenase, subunit 1	12781	16999	27120	11043	14704
13	MT-ND4	Mitochondrial NADH dehydrogenase, subunit 4	10230	12277	22878	9173	10553
14	TMSB10	Thymosin Beta 10	4811	11940	13543	4543	11381
15	MT-ND4L	Mitochondrial NADH dehydrogenase, subunit 4L	9828	11180	23128	9677	10315
16	MT-ND2	Mitochondrial NADH dehydrogenase, subunit 2	7796	10315	15025	7448	9293
17	RPS2	Ribosomal Protein S2	4152	10111	12917	3841	6642
18	IAV-PB1	IAV polymerase PB1	0	9984	26035	0	9004
19	IAV-NA	IAV neuraminidase	0	7904	5465	0	8866
20	MT-ND3	Mitochondrial NADH dehydrogenase, subunit 3	3346	7129	11255	2608	5471
22	IAV-PB2	IAV polymerase PB2	0	6407	12052	0	5737
78	IAV-PA	IAV polymerase PA	0	2609	3645	0	2909

*Ranked by average TPM of H1-8.

Table S2. The enriched functional classes upon IAV infection by GO in H1 and P.

Functional Class	GO Biological Process Term	H1-8/H1-0			P-8/P-0		
		#DEG/ #In class	Fold Enrichment	FDR	#DEG/ #In class	Fold Enrichment	FDR
**Upregulated**							
Inflammatory Response	Inflammatory response	67/266	2.13	<0.001	65/266	2.37	<0.001
G-protein	Adenylate cyclase-modulating G protein-coupled receptor signaling pathway	45/180	2.11	0.008	43/180	2.31	0.002
**Downregulated**							

DNA Replication	DNA strand elongation involved in DNA replication	8/14	6.27	0.007	8/14	8.9	0.001
	DNA replication	52/132	4.32	<0.001	42/132	4.96	0.000
	Negative regulation of cell cycle phase transition	30/108	3.05	<0.001	20/108	2.89	0.003
	Double-strand break repair via homologous recombination	22/87	2.77	0.003	20/87	3.58	0.000
Protein Processing and Trafficking	Protein targeting to lysosome	8/16	5.48	0.013	6/16	5.84	0.037
	Protein localization to Golgi apparatus	9/25	3.95	0.032	9/25	5.61	0.005
	Golgi to plasma membrane transport	14/42	3.66	0.005	11/42	4.08	0.009
	Protein localization to endoplasmic reticulum	17/59	3.16	0.005	-	-	-
	Regulation of response to endoplasmic reticulum stress	16/70	2.51	0.046	-	-	-
Lipid Metabolism	Fatty-acyl-CoA metabolic process	9/20	4.94	0.011	-	-	-
	Very long-chain fatty acid metabolic process	10/29	3.78	0.024	8/29	4.3	0.033
RNA Processing	snRNA processing	9/24	4.11	0.026	-	-	-
	Regulation of transcription elongation from RNA polymerase II promoter	9/24	4.11	0.026	7/24	4.54	0.047
	mRNA export from nucleus	12/45	2.93	0.044	-	-	-
	Nuclear-transcribed mRNA catabolic process	21/86	2.68	0.008	15/86	2.72	0.027
	mRNA catabolic process	24/107	2.46	0.006	18/107	2.62	0.015
Cell Growth	Regulation of transforming growth factor beta production	10/28	3.92	0.020	-	-	-
	Cell growth	-	-	-	13/65	3.12	0.020

#DEG: number of differentially expressed gene enriched in the comparison. #In class: number of genes in the gene set. FDR: false discovery rate.

Table S3. The enriched functional classes upon IAV infection by GSEA in H1 and P.

Functional Classes	KEGG Terms		H1-8/H1-0		P-8/P-0		H1-16/H1-8	
		SIZE	NES	FDR	NES	FDR	NES	FDR
**Upregulated**								
Ribosome	Ribosome	82	2.44	<0.001	2.67	<0.001	2.27	<0.001
**Downregulated**								
DNA Replication	DNA replication	35	−2.66	<0.001	−2.50	<0.001		
	Mismatch repair	22	−2.22	<0.001	−2.27	<0.001		
	Base excision repair	31	−2.02	0.004	−1.96	0.004		
	Homologous recombination	25	−1.97	0.005	−2.10	0.001		
	Nucleotide excision repair	43	−1.90	0.008	−1.66	0.038		
RNA Processing	Spliceosome	117	−2.05	0.003	−1.75	0.025		
	RNA degradation	55	−1.67	0.036	−1.73	0.028		
Energy Metabolism	Citrate cycle TCA cycle	28	−2.20	0.001	−2.07	0.001	−1.94	0.003
	Pentose phosphate pathway	19	−2.11	0.002	−1.91	0.007	−2.04	0.003
	Glycolysis gluconeogenesis	38	−1.83	0.013	−2.21	<0.001	−1.73	0.019
	Fructose and mannose metabolism	26	−1.71	0.028			−1.76	0.017
Lipid Metabolism	Sphingolipid metabolism	31					−1.68	0.031
	Butanoate metabolism	21					−1.76	0.016
	Propanoate metabolism	26			−1.78	0.020		
	Endocytosis	134	−2.01	0.004	−1.94	0.006	−2.03	0.002
	Lysosome	105	−1.87	0.009			−1.63	0.046

Protein Processing and Trafficking	Ubiquitin mediated proteolysis	121	−1.63	0.042	−1.67	0.039
Others	Antigen processing and presentation	31	−1.76	0.021		
	Regulation of autophagy	18	−1.74	0.022		
	Cell cycle	119	−2.28	0.001	−2.35	<0.001

SIZE: number of differentially expressed gene enriched in the comparison. NES: normalized enrichment score. FDR: false discovery rate.

Table S4. The enriched gene sets by GSEA in H1-0/P-0 and H1-8/P-8 comparison.

Functional Class	GO biological process term	SIZE	H1-0/P-0 NES	H1-0/P-0 FDR	H1-8/P-8 NES	H1-8/P-8 FDR
	**Positively Enriched**					
RNA processing	Nuclear transcribed mRNA catabolic process NMD (nonsense mediated decay)	112	2.23	<0.001	2.23	0.001
	rRNA metabolic process	232	-	-	2.19	0.002
Ribosome	Ribosome biogenesis	278	2.50	<0.001	2.00	0.018
Translation	Translational termination	87	2.65	<0.001	2.11	0.004
	Translational initiation	132	2.36	<0.001	1.94	0.031
	Translational elongation	101	2.52	<0.001	-	-
	Mitochondrial translation	100	2.64	<0.001	2.07	0.007
Mitochondrion and Energy Metabolism	Oxidative phosphorylation	63	2.27	<0.001	2.16	0.003
	Mitochondrial translation	100	2.64	<0.001	2.07	0.007
	Mitochondrial respiratory chain complex assembly	63	2.25	<0.001	-	-
	Electron transport chain	72	2.09	0.002	-	-
DNA Replication	DNA replication	181	2.08	0.003	-	-
	DNA strand elongation	29	1.96	0.01	-	-
	**Negatively Enriched**					
Antiviral Response	Response to type I interferon	33	-	-	-2.31	<0.001
	Defense response to virus	90	-	-	-2.27	<0.001
	Response to virus	146	-	-	-1.90	0.035
	Negative regulation of viral genome replication	26	-	-	-1.90	0.037
	Immune effector process	248	-	-	-1.89	0.039
Protein Trafficking	Endocytosis	305	-	-	-1.99	0.02
	Golgi vesicle transport	262	-	-	-2.16	0.001
	ER to golgi vesicle mediated transport	134	-	-	-1.94	0.025

SIZE: number of differentially expressed gene enriched in the comparison. NES: normalized enrichment score. FDR: false discovery rate.

Table S5. Representative differentially expressed genes in antiviral responses.

Gene Symbol	Description	Average TPM H1-0	H1-8	H1-16	P-0	P-8
RIG-I	DDX58: DExD/H-box helicase 58	77.77	113.1	98.55	94.33	149.7
MDA5	IFIH1: Interferon Induced With Helicase C Domain 1	101.8	32.02	18.76	104.2	53.34
TRIM25	tripartite motif containing 25	95.37	52.27	37.93	107.8	73.81
MAVS	mitochondrial antiviral signaling protein	7.82	9.37	8.95	11.30	13.42
TLR3	toll like receptor 3	35.56	13.11	7.36	36.64	21.50

MYD88	MYD88 innate immune signal transduction adaptor	199.0	253.3	361.3	206.4	317.9
IRF7	Interferon Regulatory Factor 7	73.09	63.21	33.41	62.45	99.83
NFKB1	nuclear factor kappa B subunit 1	38.75	24.36	18.33	41.43	30.39
NFKB2	nuclear factor kappa B subunit 2	31.63	15.39	6.62	25.39	19.33
RELB	RELB proto-oncogene, NF-kB subunit	11.94	26.91	21.83	9.04	20.58
RELA	RELA proto-oncogene, NF-kB subunit	78.66	52.59	41.10	75.81	63.50
REL	REL proto-oncogene, NF-kB subunit	10.69	6.97	6.79	13.29	8.06
NFKBIB	NFKB inhibitor beta	74.92	81.37	63.83	64.56	72.28
NFKBID	NFKB inhibitor delta	1.52	6.62	4.49	2.00	4.39
NFKBIE	NFKB Inhibitor Epsilon	8.78	21.83	11.57	6.75	13.62
NFKBIL1	NFKB inhibitor like 1	20.24	34.51	34.88	16.89	26.12
TYK2	tyrosine kinase 2	31.18	15.88	9.02	37.52	22.02
JAK1	Janus kinase 1	124.4	44.74	29.39	150.7	65.53
STAT1	signal transducer and activator of transcription 1	120.1	50.47	38.43	147.9	78.67
STAT2	signal transducer and activator of transcription 2	72.81	27.97	21.75	89.16	44.24
STAT3	signal transducer and activator of transcription 3	73.99	30.10	14.29	100.2	49.22
IRF9	Interferon Regulatory Factor 9	45.93	36.69	21.28	54.90	51.06
IFNE	Interferon Epsilon	2.45	1.88	1.82	3.69	4.14
IFIT3	interferon induced protein with tetratricopeptide repeats 3	2.57	10.18	16.34	11.35	48.23
OAS1	2'-5'-oligoadenylate synthetase 1	256.3	183.1	144.7	327.5	350.8
OAS2	2'-5'-oligoadenylate synthetase 2	28.90	14.86	16.46	72.58	55.81
OAS3	2'-5'-oligoadenylate synthetase 3	65.99	49.41	25.11	104.5	89.37
MX1	MX dynamin like GTPase 1	128.6	116.2	112.8	250.5	328.4
MX2	MX dynamin like GTPase 2	17.72	7.36	4.73	61.91	66.43
ISG15	ISG15 ubiquitin like modifier	821.9	919.9	706.0	1088	1848
ISG20	interferon stimulated exonuclease gene 20	20.18	28.13	21.60	30.34	51.65
DDX60	DExD/H-box helicase 60	45.23	16.67	13.26	90.93	46.38
IRF1	interferon regulatory factor 1	138.7	91.95	60.42	95.97	66.40
IFIT1	LOC488947: Interferon Induced Protein With Tetratricopeptide Repeats 1	2.64	11.82	23.70	7.29	32.82
SAMHD1	SAM and HD domain containing deoxynucleoside triphosphate triphosphohydrolase 1	18.21	6.93	4.98	28.01	17.56
RSAD2	radical S-adenosyl methionine domain containing 2	146.8	336.8	166.3	250.8	707.8
BST2	bone marrow stromal cell antigen 2	323.2	217.0	225.2	454.4	376.0
SPON2	Spondin 2	0.99	0.50	0.75	4.15	6.55
CD40	CD40 molecule	9.30	5.16	2.58	5.93	3.48
IRF5	interferon regulatory factor 5	9.06	10.26	6.95	7.33	7.21
IFIT2	interferon induced protein with tetratricopeptide repeats 2	1.50	7.21	15.10	2.63	18.11
CD86	CD86 molecule	2.67	5.01	4.76	4.63	9.28
RNASEL	ribonuclease L	36.38	16.76	9.81	34.48	25.11

SERINC3	serine incorporator 3	22.88	8.78	6.68	27.43	15.00
IFI44L	interferon induced protein 44 like	153.1	75.88	34.46	176.6	143.1

Table S6. Raw data of all the gene expression (Normalized Counts, Average TPM) and DEseq2 output. See Supplementary File 2 Table S6.xlsx.

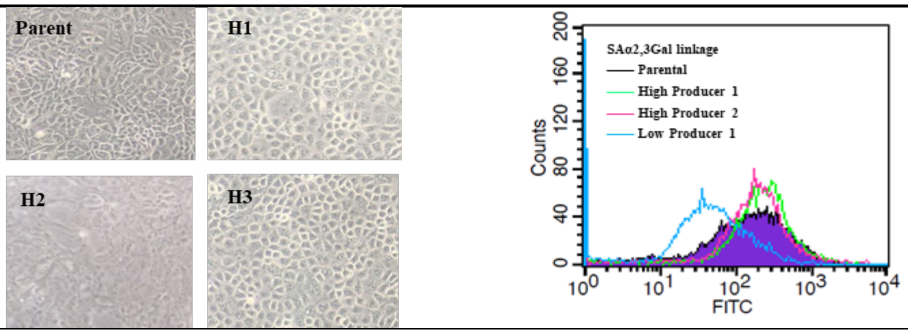

(**a**) (**b**)

Figure S1. (**a**): Morphology of parental cells and high producing clones. (**b**): flowcytometric assay of α-2,3 SA receptor in the H1, H2, L1, and parental cells (P).

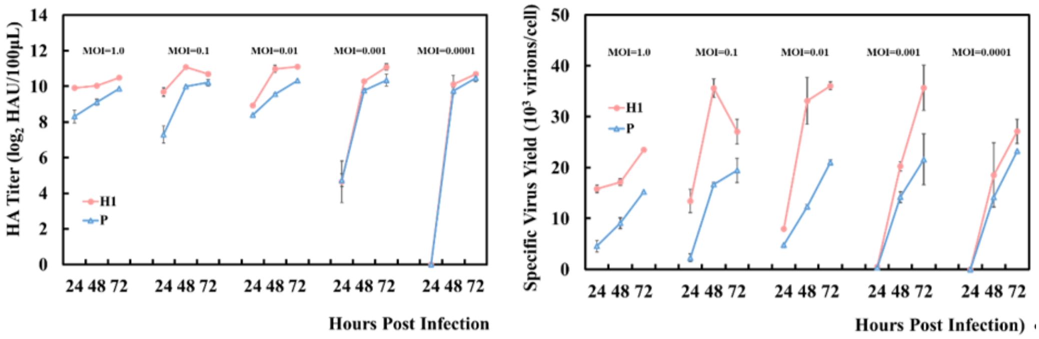

(**a**) (**b**)

Figure S2. H1 has higher virus productivity at different MOIs. (**a**): HA titer and (**b**): specific virus yield at different MOIs from 24-72 hpi. Solid red: H1, hollow blue: P.

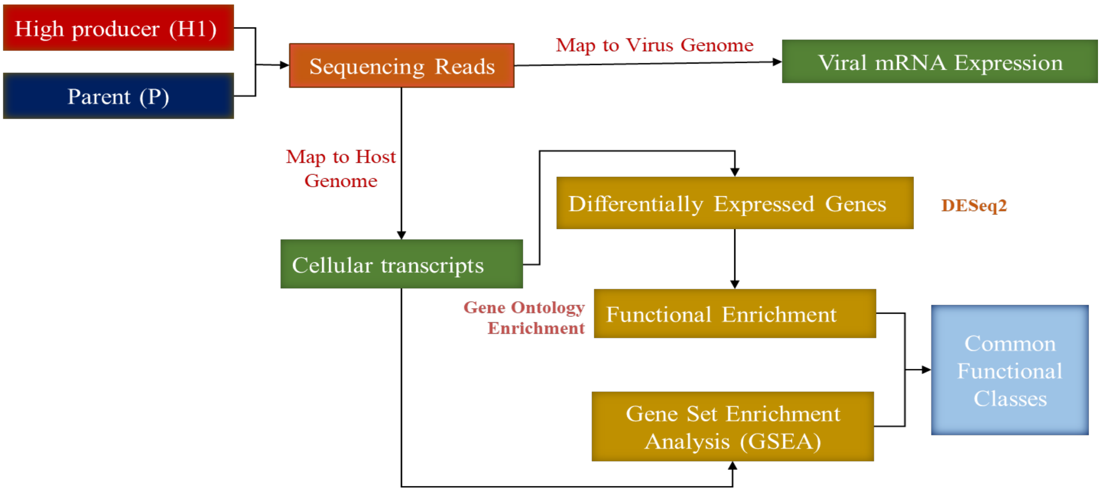

Figure S3. Transcriptome analysis pipeline.

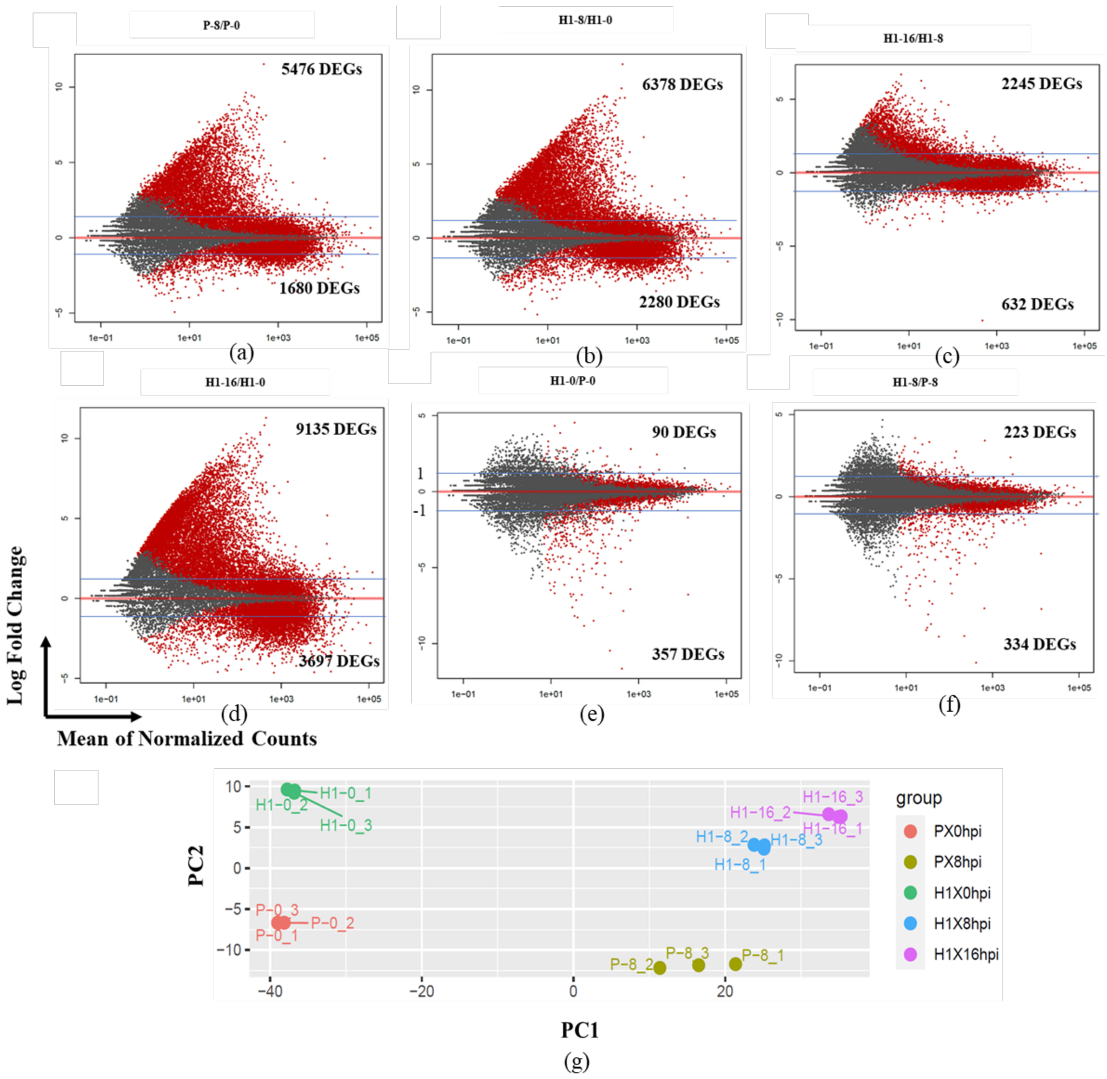

Figure S4. Pair−wise comparison gene expression**.** DESeq2 was applied to identify the differentially expressed genes (DEGs) of (**a**) H1−0/P−0, (**b**) H1−8/P−8, (**c**) H1−16/H1−8, (**d**) P−8/P−0, (**e**) H1−8/H1−0, (**f**) H1−16/H1−0, Red represents transcripts with p_{adj}<0.05. (**g**): Principal Components Analysis of the transcriptome of H1 and parent at 0/8/16 hpi in the synchronized single cycle infection. Uninfected and infected samples are separated along PC1, while samples from H1 or P are separated by PC2.

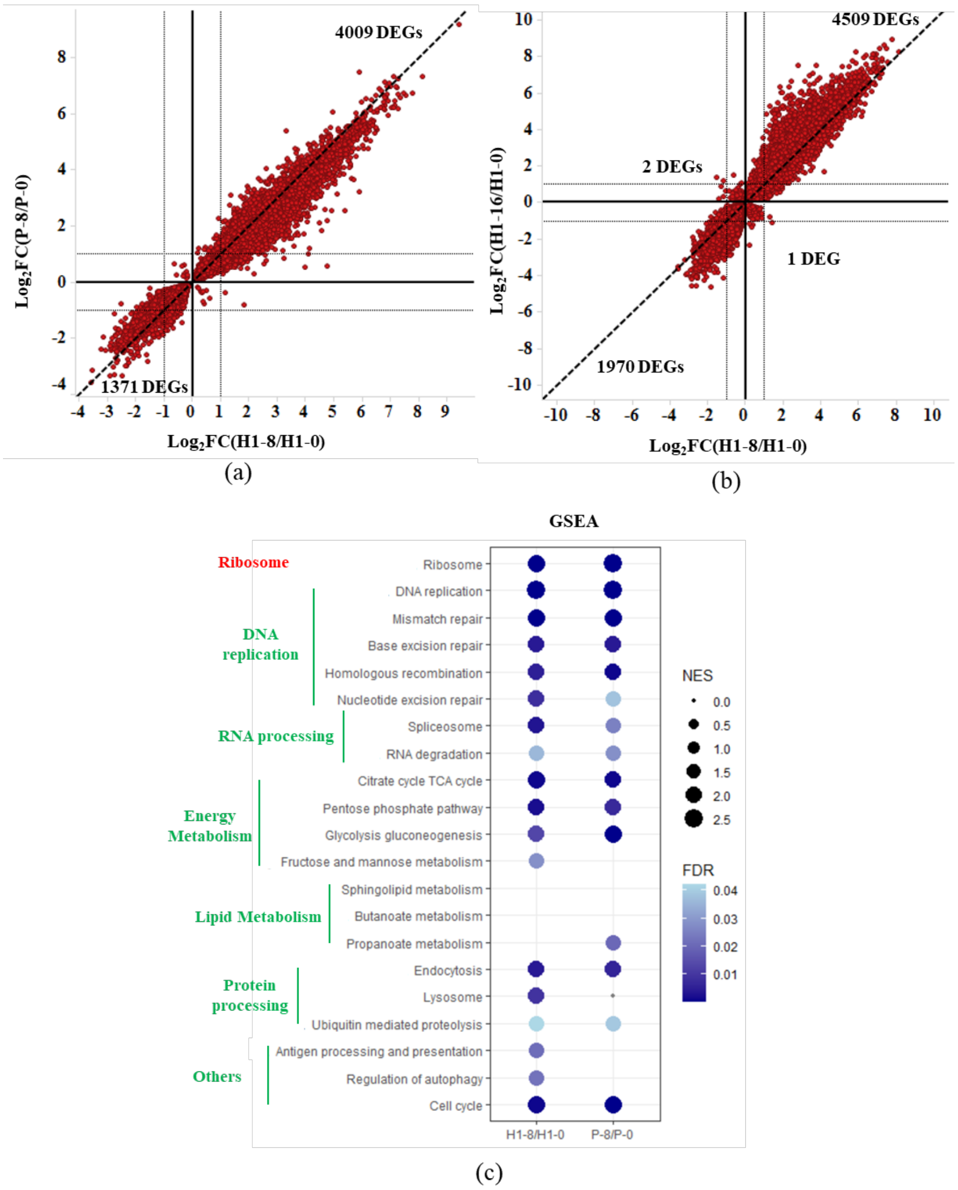

Figure S5. Comparison of response upon virus infection in H1 and P. (**a**): comparison of response between 8 hpi and 0 h in H1 and P. (**b**) comparison of response of H1 between 8 hpi and 0 h, and between 16 hpi and 0 h. (Dot represents the genes with p_{adj} <0.05 in both pair of comparison. The dotted lines mark the fold change of 2 and the black dash line represents equal FC in both pairs of comparison.) (**c**): GSEA on host response of H1 and P upon IAV infection, dot size represents the normalized enrichment score, dot color represents the FDR. Colors of clusters GO terms represents direction of change – red: up-regulated, green: down-regulated.
